# Quality of life and mental health in the locked-in-state—differences between patients with amyotrophic lateral sclerosis and their next of kin

**DOI:** 10.1007/s00415-022-11238-0

**Published:** 2022-07-06

**Authors:** Elisa Aust, Katharina Linse, Sven-Thomas Graupner, Markus Joos, Daniel Liebscher, Julian Grosskreutz, Johannes Prudlo, Thomas Meyer, René Günther, Sebastian Pannasch, Andreas Hermann

**Affiliations:** 1 Department of Neurology, Technische Universität Dresden, Dresden, Germany; 2 Deutsches Zentrum für Neurodegenerative Erkrankungen (DZNE) Dresden, Dresden, Germany; 3 Engineering Psychology and Applied Cognitive Research, Technische Universität Dresden, Dresden, Germany; 4 Interactive Minds Research, Interactive Minds Dresden GmbH, Dresden, Germany; 5 Precision Neurology, University of Lübeck, Lübeck, Germany; 6 Department of Neurology, University of Rostock, Rostock, Germany; 7 Deutsches Zentrum für Neurodegenerative Erkrankungen (DZNE) Rostock/Greifswald, Rostock, Germany; 8 Center for ALS and other Motor Neuron Disorders, Charité – Universitätsmedizin Berlin, Corporate Member of Freie Universität Berlin, Humboldt-Universität zu Berlin, and Berlin Institute of Health, Berlin, Germany; 9 Center for Transdisciplinary Neurosciences Rostock (CTNR), University Medical Center Rostock, University of Rostock, Rostock, Germany; 10 Translational Neurodegeneration Section “Albrecht Kossel,” Department of Neurology, University Medical Center Rostock, University of Rostock, Gehlsheimer Straße 20, 18147 Rostock, Germany

**Keywords:** Eye tracking, Quality of life, Anxiety, Caregiver burden, Palliative care, End of life decisions

## Abstract

For both patients with amyotrophic lateral sclerosis (ALS) and their next of kin (NOK), the maintenance of quality of life (QoL) and mental health is particularly important. First studies suggest significant discrepancies between QoL reports by patients and NOK, but little is known for advanced ALS stages. To address this issue, we screened 52 ALS patients in incomplete locked-in state (iLIS). Final results were obtained for 15 couples of iLIS patients and NOK. We assessed patients’ and NOK’s subjective QoL, depression and anxiety and NOK’s caregiver burden. Gaze controlled questionnaires allowed direct assessment of patients. Patients and NOK self-reported comparable, mostly moderate to high levels of QoL. Of note, NOK indicated stronger anxiety symptoms. Higher anxiety levels in NOK were associated with stronger caregiver burden and reduced QoL. No significant misjudgment of patient’s QoL by the NOK was evident, while patients overestimated NOK’s global QoL. However, NOK with severe caregiver burden and depression symptoms gave poorer estimations of patients’ QoL. This relationship is relevant, considering NOK’s impact on life critical treatment decisions. While the daily time NOK and patient spend together was positively correlated with NOK’s QoL and mental health, this was not reversely found for the patients. Our results suggest that NOK adapt less successfully to the disease and concomitant experience of loss and point to an urgent need for specialized psychosocial support. The findings emphasize the importance of direct psychological wellbeing assessment of both patients and NOK in clinical practice, enabled by eye-tracking technology for patients in iLIS.

**Supplementary Information:**

The online version contains supplementary material available at 10.1007/s00415-022-11238-0.

## Introduction

Amyotrophic lateral sclerosis (ALS) is characterized by the degeneration of motor neurons, resulting in the progressive and fatal loss of voluntary muscle control. It can progress into an incomplete locked-in state (iLIS) in which patients are completely paralyzed, immobile and no longer capable to communicate verbally, while being fully conscious and able to control their eyes [1, 2]. Communication in iLIS is therefore enabled by eye tracking communication devices (ETCS). Most ALS-patients in iLIS depend on invasive ventilation (IV), which extends lifetime in average up to 11 years [3]. The majority of ALS patients who reject IV die within 2–5 years after the onset of symptoms [4].

Since ALS is not curable, care and treatment at every stage of the disease aims at maintaining patients’ QoL and psychological wellbeing [5, 6]. QoL and mental health are obviously seriously threatened by the symptoms and prognosis of the disease, which results in a loss of autonomy by restricting social interaction and participation in all areas of life. It is therefore not surprising that ALS patients’ *health related* QoL— which per definition is determined by the degree of physical functioning, mobility, independence etc.— declines as the disease progresses [7–12]. However, in a number of studies ALS patients expressed a moderate or even high *subjective* QoL [13–16]. Their subjective QoL (hereafter referred to by “QoL”) even remained stable over the course of the disease despite the severe and worsening physical impairment [10, 15, 17–19]. It has also been found to be similar in patients with and without IV [1, 16, 17, 20]. Poorer QoL in ALS patients is associated with stronger symptoms of depression [17, 18, 21] and anxiety [9, 22], but those symptoms reach a clinically relevant level in only about 10–30% of the patients [18, 21–23].

However, QoL-research in ALS so far is focused on patients in early and moderate disease stages who can still be investigated by means of spoken or written language. Only very few studies include advanced ALS patients [24–26]. We were the first to assess iLIS patients’ QoL directly and thus independent from their NOK by means of a battery of questionnaires controlled by gaze using an ETCS [24]. Patients in this previous study reported a very high QoL, in contrast to a much lower NOK’s QoL. The stable positive QoL in patients with ALS suggests the existence of an effective adaption process. Apparently, they manage to readjust their expectations and value a satisfying life that is worth living [17, 26–28]. Social relationships and support appear as the most important psychological resource in this process in earlier ALS stages [5, 13, 17] and also in iLIS [24].

At the same time, ALS patients’ NOK often report high levels of strain. They experience a loss of their way of life and future plans due to the disease and to patients’ increasing dependence on support and care. Furthermore, NOK caregiver burden increases [29–33] and their psychological wellbeing and mental health decreases with the decline of the ALS patients’ functional status over time, while remaining stable in the patients themselves [32–34]. In direct comparison of NOK and patients, NOK indicate a similar [33–36] or even lower QoL [15, 24, 33]. Again though, only few of those studies look at NOK of advanced ALS patients. In our previous iLIS-study [24], NOK’s lower own QoL was accompanied by their underestimation of patients’ QoL. Such an underestimation of ALS patients’ wellbeing and QoL by their relatives has been shown before [1, 25, 26]. Considering the influential role of NOK in decision making—particularly over initiation and termination of life-prolonging measures [37–39]—those misjudgments can have tremendous impact on iLIS patients’ safety and the fulfillment of their will.

The overall and first aim of the present study was to assess QoL in iLIS patients and their NOK, regarding how their self- and external evaluations differ and interact. In order to gain a better understanding of QoL and these expected differences and interactions, we took a closer look at the areas of life that patients and NOK report as important for their QoL. The second aim was to examine relationships between symptoms of depression, anxiety, caregiver burden and QoL. We specifically assessed if NOK’s judgment of iLIS patients QoL might be influenced by NOK’s own wellbeing and experienced burden. Third, we tried to identify factors that might have an impact on iLIS patients’ and NOK’s wellbeing by analyzing sociodemographic and disease related characteristics together with QoL, mental health and caregiver burden. Additionally, patients’ attitude toward of IV as the most important life-prolonging measure was investigated. An ETCS-based version of the battery of questionnaires allowed for assessing iLIS patients directly and completely independent from their NOK.

## Methods

### Sample recruitment

For our cross-sectional observation study, patients and NOK were recruited as a convenience sample from specialist outpatient clinics at the University Hospital in Dresden, the Charité Berlin, the University Hospitals in Rostock, Jena, Goettingen, Hannover, and from a patient network (ALS mobil e.V.).

Inclusion criteria were an established ALS according to El Escorial criteria [40] or an ALS variant (primary lateral sclerosis, PLS; progressive muscular atrophy, PMA) and presence of iLIS (tetraplegia and very severe dysarthria or anarthria with loss of mobility, preserved eye movements, at most minimal residual head or limb movement, e.g., of toes, individual fingers, face muscles). Exclusion criteria were a severe horizontal or vertical gaze palsy, a clinically defined frontotemporal dementia or obvious severe cognitive impairment, evaluated by two psychologists with significant experience with ALS and iLIS patients.

### Measures

Disease severity was quantified by the *ALS Functional Rating Scale-Revised* (ALSFRS-R) [41]. The scale consists of 12 items and results in a single score with a range from 0 (complete loss of movement, anarthria and dependence of IV and invasive nutrition) to 48 (normal motor abilities).

For all following questionnaires, we implemented ETCS-based versions in order to enable iLIS patients to complete them independently. For the assessment of QoL, the *McGill-SIS Quality of Life Single Item Scale* (McGill-SIS) and the *Schedule for the Evaluation of Individual Quality of Life-Direct Weighting* (SeiQoL-DW) were used. The McGill-SIS [42] requires subjects to rate their global QoL on a 11-point Likert-scale from 0 (worst possible QoL) to 10 (best possible QoL). For the SeiQoL-DW as a measure of subjective QoL, we used a version with a list of areas of life from which subjects first selected the five they consider most important for their current QoL [43]. For each of the five selected areas they had to indicate the subjective importance and to rate the subjective degree of satisfaction on an 11-point Likert-Scale from 0 to 100 (steps of ten, 0 = not satisfied at all, 100 = perfectly satisfied), respectively. The SeiQoL-Score Index as parameter of QoL was then calculated as the sum of those satisfaction ratings weighted by the importance ratings (see [44] for exact calculation method). The SeiQoL-DW-Score Index ranges from 0 to 100, with higher values representing a better subjective QoL.

Depressive and anxiety symptoms were measured by means of the *Hospital Anxiety and Depression Scale* (HADS), a questionnaire particularly developed for assessing those symptoms in patients with somatic disorders [45]. The HADS consists of two subscales, each ranging from 0 to 21 points. For both subscales, scores from 8 to 10 indicate mild symptoms, scores from 11 to 14 moderate and scores ≥ 15 severe symptoms of depression or anxiety. Since scores ≥ 11 (moderate to severe symptoms) are classified as valid cases of depression (versus doubtful cases) also in studies of ALS patients [46], we used this cut-off for classification and regarding subgroup comparisons. All patients with IV were asked if they would decide for IV again, which they answered with “yes” or “no.”

All iLIS patients answered self-rating versions of the questionnaires and an additional external rating-version of the McGill-SIS to evaluate the QoL of their NOK.

All NOK completed the equivalent paper–pencil-versions of the questionnaires. They completed both the McGill-SIS and SeiQoL-DW in self-rating as well as in an external rating-version to evaluate the QoL of the patients.

Additionally, the *Burden Scale for Family Caregivers*, short version (BSFC-s) was used to quantify NOK caregiver burden resulting from (nursing) care and/or support of their relative with ALS [47]. Scores in the BSFC-s range from 0 to 30 points, with higher scores indicating stronger caregiver burden. Scores under 10 are classified as low, scores from 10 to 20 as moderate and scores from 21 to 30 as severe caregiver burden.

### Testing procedure

A monocular Eyegaze Edge^®^ remote infrared eye-tracking device with a sampling rate of 50 Hz (LC Technologies) allowed the iLIS patients to interact by gaze and was used for the data collection. Patients were either lying in bed or sitting in a wheelchair, with the ETCS-screen positioned fronto-parallel in a distance of 60–70 cm to the face. The protocol started with a 9-point calibration, followed by the battery of questionnaires and tests. An additional observer screen was used to control for a reliable gaze control of the ETCS and to make sure that iLIS-patients read the instructions. The iLIS-patients then completed the questionnaires autonomously and without active interventions by the experimenter. An exception was the SeiQoL-DW: because of its complexity, the experimenter observed iLIS patients answering behavior and intervened in cases in which patients presented difficulties that might lead to unintended or unreliable answers.

NOK were interviewed in parallel to patients’ assessment in a separate room by a second experimenter and based on a protocol developed for the study. During the interviews, sociodemographic and (objective) information regarding the ALS disease, care and life situation were collected. All NOK completed paper–pencil versions of the questionnaires on their own, either directly after the interview or within eight days after the study visit. All study visits took place at patients’ and/or NOK’s home or in the nursing home where the patient lived. Neither patients nor NOK were informed about the results of their respective significant others.

### Statistical analysis

Statistical analyses were conducted in R [Version 1.2.5, 48]. We used Wilcoxon signed rank test for continuous data and McNemar’s test for categorical data to compare the characteristics and ratings between patients and their NOK (paired samples). For comparisons between different subgroups of patients or NOK, we conducted Mann–Whitney *U*-Test for independent samples. Correlations were determined using Spearman rank correlation coefficient. The significance level was set at *p* < 0.05 (two-tailed tests). Since non-parametric tests were used, results are displayed as median (Mdn) and interquartile range (IQR: quartile 1–quartile 3).

## Results

### Subjects

**Fig. 1:**
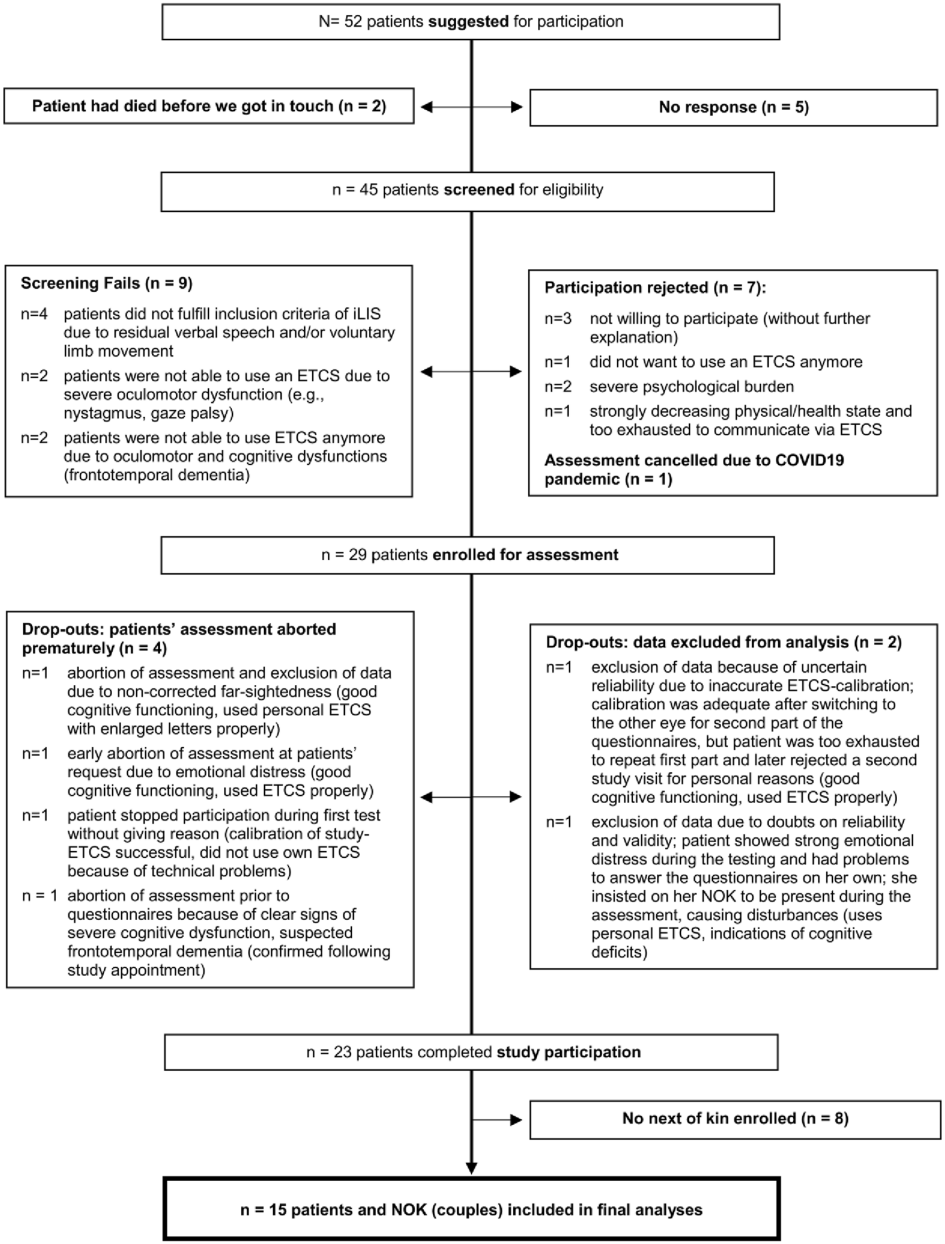
Flowchart of sample recruitment

Fifty-two suspected LIS patients with ALS or ALS-variants were suggested for study participation by the clinics or patient network; they either contacted us or we contacted them. From the overall sample, five patients did not respond and two patients had already passed away at the time we approached them. The remaining 45 patients were screened for eligibility. Seven patients decided not to participate after the screening and ten patients did not meet the criteria for participation (e.g., no iLIS, see Fig. 1). One patient could not be assessed due to begin of COVID19-pandemic. Therefore, we enrolled 27 patients in the study. In case of four patients, we stopped the assessment prematurely and/or excluded the collected data (Fig. 1 for reasons). The remaining 23 patients completed the respective study procedures successfully. For eight of those patients, no NOK participated in the study.

Consequently, 15 ALS-patient-NOK-pairs were included in the final analyses. Their demographic and clinical characteristics are shown in Table 1. The respective data for the excluded patients (with missing data) is displayed in table S.1 (Supplement). Of the ten patients who lived at home, seven patients received a 24-h outpatient nursing service, one patient received a 22-h outpatient nursing service, one was provided by a 13-h outpatient nursing service and additional family care and one was cared for by his spouse only. All 15 patients have been supplied with a personal ETCS (from different manufactures) and were experienced users of such systems. Four patients did not use invasive or non-invasive ventilation (ALS-FRS range: 10–18), but were tetraplegic and thus immobile and had functional or full anarthria. All NOK were patients’ spouses or life partners. In two cases the spouse was also part of the patients’ professional nursing team.

**Table 1:** Demographic and clinical characteristics and results from the psychosocial questionnaires for all participants included in the final analysis: *n* = 15 couples

Characteristic	iLIS patients	NOK	*p *value^b^
Gender, Female:Male, %	34:66	60:40	0.302
Age, year^a^	56.0 [52.5–61.0]	56.0 [50.5–59.0]	0.662
Married/in partnership, %	100	100	1.00
Education, year^a^	18.0 [16.0–18.0]	13.0 [13.0–17.5]	0.068
Working activity, %			
Employed:Retired or unemployed	6.7:93.3	86.7:13.3	< 0.001*
Place of living, Home:Nursing home, %	67.7:33.3	100:0	0.050
Antidepressant medication, %	46.7	6.7	< 0.001
ALS onset, bulbar:spinal, %	46.7:53.3	–	–
ALS duration, year^a^	5.6 [3.4–7.7]	–	–
ALSFRS-R^a^	1 [0–6]	–	–
IV, %; duration, year^a^	73.3; 2.1 [0.25 – 4.3]	–	–
ETCS-use, duration, year; use h/day^a^	2.2 [0.7–3.2]; 11 [5.5–14.5]	–	–
McGill-SIS-Score			
Self-rated^a^	4.0 [3.0–8.0]	5.0 [3.0–6.5]	0.949
External rating for the respective other party^a^	5.0 [3.0–6.0]	6.0 [5.0–8.0]	0.85^c^/0.012^d*^
SeiQoL-DW-Index			
Self-rated^a,f^	66.1 [50.4–79.3]	64.9 [57.9–76.9]	0.100
External rating by NOK^a,f^	57.6 [40.8–69.3]	–	0.295^c^
HADS: Depression			
Score^a^	6.0 [4.5–9.5]	7.0 [5.0–13.0]	0.266
Severity: moderate or severe	20	33.3	0.632/0.617
Under cut-off or mild, %	80	67.7	
HADS: Anxiety			
Score^a^	7.0 [4.0–9.0]	8.0 [6.5–14.0]	0.015*
Severity: moderate or severe	6.7	46.6	0.052/.041*
Under cut-off or mild, %	93.3	53.4	
BSFC-*k*-Score	-	15.0 [11.5–25.0]	–

*NOK* next of kin, *ALS* amyotrophic lateral sclerosis, *ALSFRS-R* ALS Functional Rating Scale Revised, *IV* invasive ventilation, *ETCS* eye tracking computer system, *McGill-SIS* McGill Quality of Life Single Item Scale, *SeiQoL-DW* Schedule for the Evaluation of Individual Quality of Life-Direct Weighting, *HADS* Hamilton Anxiety and Depression Scale, *BSFC-s* Burden Scale for Family Caregivers - short version

^*^Statistically significant (*α* = 5%)

^a^Data presented as median [interquartile range: Q1–Q3]

^b^McNemar-test or Wilcoxon signed rank test for paired samples

^c^For comparison between patients’ self-rating and rating for patients by NOK

^d^For comparison between NOK’s self-rating and rating for NOK by patients

^f^Data available for *n* = 14 couples

### Level of QoL

#### Comparison of self-rated QoL between patients and NOK

**Fig. 2:**
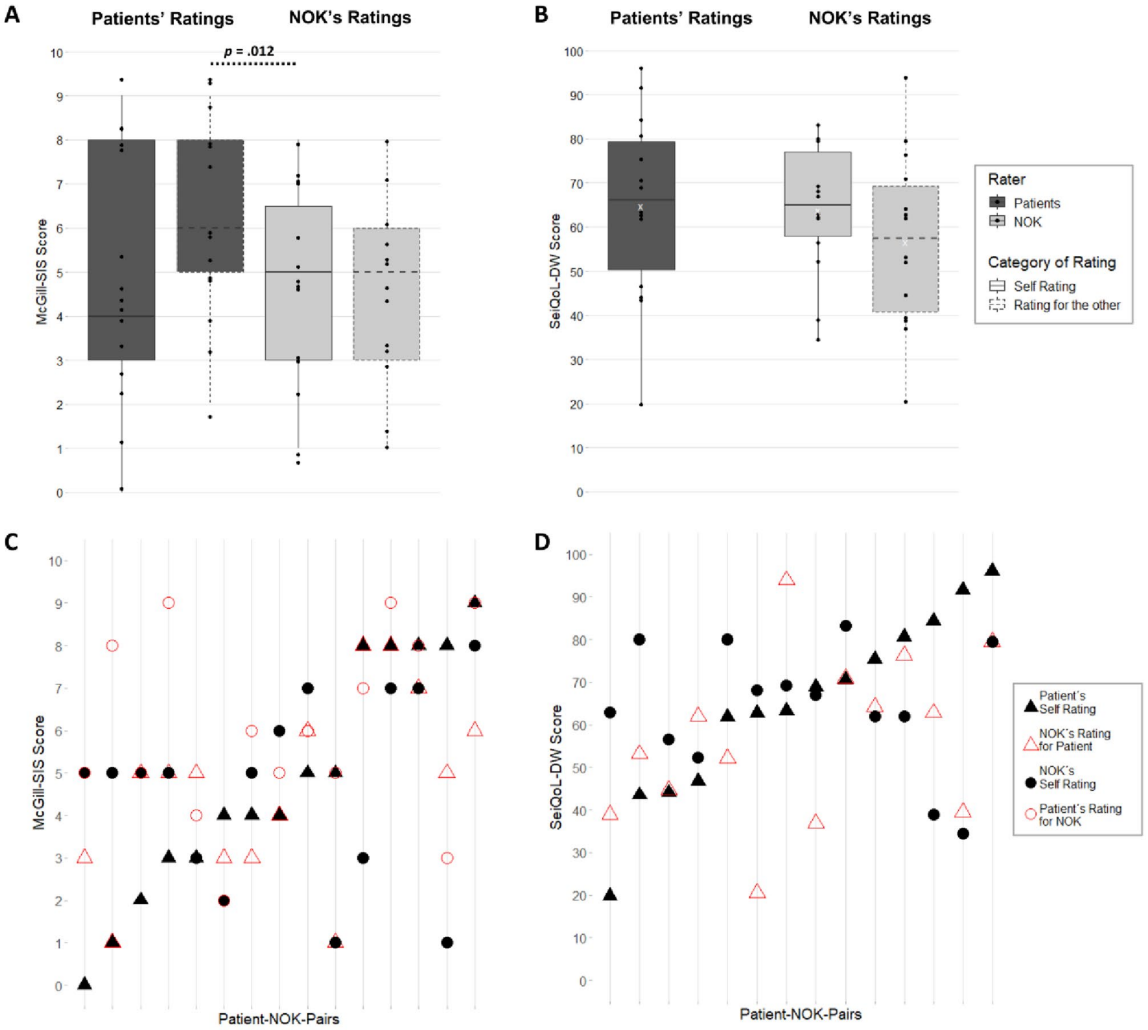
Comparison of QoL-ratings between patients and NOK in SeiQoL-DW and McGill-SIS. On group level for **A** McGill-SIS and **B** SeiQoL-DW; dots represent the individual ratings by patients/NOK; statistically significant results (*p* < 0.05) are reported for the comparison between groups (Wilcoxon signed rank test). Separately for each patient-NOK-couple for **C** McGill-SIS and **D** SeiQoL-DW; each line represents one couple with symbols for the individual ratings (patients estimated NOK’s QoL only in the McGill-SIS)

Self-rated QoL of iLIS patients and NOK was on a very similar level for both SeiQoL-DW and McGill-SIS, with a wider range of QoL-ratings in patients. There were no significant differences between those QoL-ratings (Table 1, Fig. 2). A comparative look at both scales revealed higher scores in the SeiQoL-DW than in the McGill-SIS for patients and NOK (Fig. 2). No correlations were found between QoL-self-ratings of patients and NOK (McGill-SIS: *r* = − 0.22, *p* = 0.42; SeiQoL-DW: *r* = 0.22, *p* = 0.46)*.*

#### Do NOK misjudge patients’ QoL?—Comparison of patient’s self-rating and NOK’s external evaluation

In the next step we tested the key hypothesis of a misjudgment of iLIS patient’ QoL by their NOK. On a group level, the estimations of patients’ QoL in SeiQoL-DW and McGill-SIS provided by NOK did not reliably differ from the respective QoL-ratings given by the patients for themselves (Table 1, Fig. 2A + B). However, the pairwise examination on descriptive level revealed considerable misjudgments (Fig. 2C + D).

For the SeiQoL-DW, the comparison of iLIS patients whose QoL was underestimated by NOK and those whose QoL was overestimated by NOK revealed, that patients in the underestimated subgroup self-rated their QoL significantly higher (*n* = 8, Mdn = 77.98, IQR = 67.33–86.09) than those in the overestimated subgroup (*n* = 6, Mdn = 45.35, IQR = 43.62–59.17; *p* = 0.013). The estimation of patients’ QoL by NOK did not significantly correlate with self-rating of the patients in the SeiQoL-DW (*r* = 0.43, *p* = 0.12).

For the McGill-SIS results, self-rated QoL of patients was also significantly higher for those patients where the NOK underestimated their QoL (n = 6, Mdn = 6.5, IQR = 4.25–8.0), compared with patients whose NOK overestimated their QoL (*n* = 5, Mdn = 3, IQR = 2.0–3.0;* p* = 0.027). A higher McGill-SIS rating of patients’ QoL by the NOK correlated significantly with a higher McGill-SIS-self-rating by the patient (*r* = 0.64, *p* = 0.009).

There were no significant correlations between NOK’s self-rating and their external rating of patients’ QoL, but a statistical trend (*p* > 0.10) for a positive correlation for the McGill-SIS (SeiQoL-DW: *r* = 0.19, *p* = 0.51; McGill-SIS: *r* = 0.48, *p* = 0.07).

With regard to the differences found in the level of QoL obtained by SeiQoL-DW and by McGill-SIS, we analyzed correlations between those ratings. In the patient group, a higher self-rating in the McGill-SIS was highly correlated with a higher self-rating in the SeiQoL-DW (*r* = 0.78, *p* = 0.001). For NOK’s self-ratings in the two questionnaires, there was a statistical trend for a positive relationship (*r* = 0.48, *p* = 0.085). NOK’s estimations of patients’ QoL in the two questionnaires did not correlate significantly (*r* = 0.41, *p* = 0.13).

#### Do patients misjudge NOK’s QoL? – Comparison of NOK’s self-rating and patients’ external evaluation

The comparison between NOK’s QoL as estimated by iLIS patients and as rated by the NOK themselves in the McGill-SIS revealed a significant overestimation of NOK’s QoL by the patients (*p* = 0.012). An overestimation was obtained in 66.7% of the couples (i.e., *n* = 10, deviance: Mdn = 2.3, IQR = 1–3.75). Only two patients underestimated their NOK’s QoL, by 1 point each (Fig. 1C). A higher rating of their NOK’s QoL by the patient was associated with a higher self-rating by the NOK (*r* = 0.68, *p* = 0.005).

### Areas of life important for QoL

**Fig. 3:**
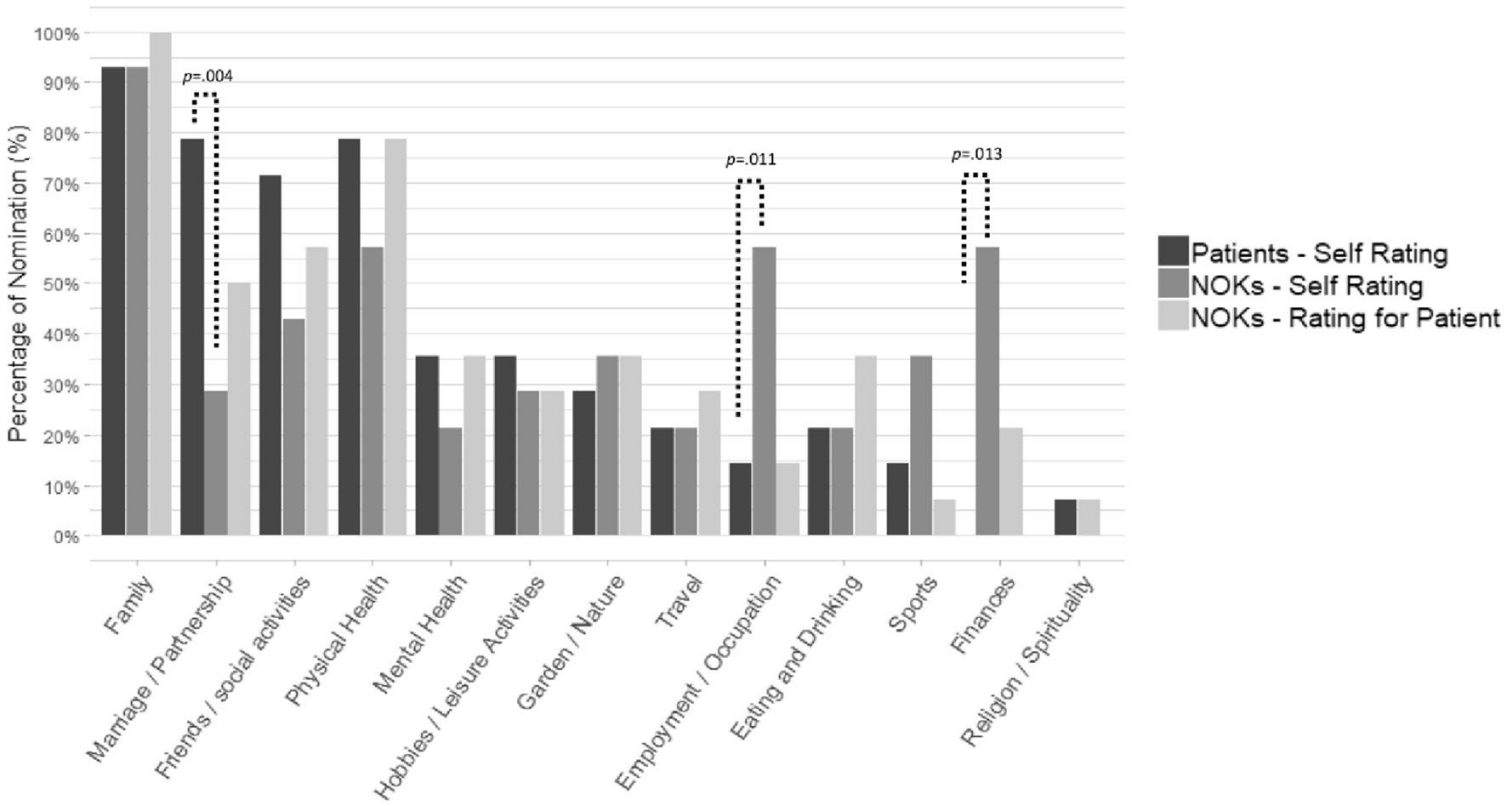
Results for chosen areas of life in the SeiQoL-DW. The figure shows the percentage of patients and NOK who nominated each area as one of the five most important ones for their current QoL. Statistically significant results (*p* < 0.05), are reported for the comparison of those frequencies (McNemar´s test)

In order to better understand differences in QoL-evaluations between iLIS patients and NOK, we compared the frequencies of the areas of life that they nominated as one of the five most important ones in the SeiQoL-DW. The analysis yielded some remarkable differences between patients and NOK as displayed in Fig. 3.

Since *Family* was the area of life most often nominated by both iLIS patient and their NOK in their self-rating of QoL (*n* = 12 couples), we directly compared those *satisfaction*-ratings. Patients reported a higher satisfaction for *Family* (Mdn = 90.0, IQR = 70–100) than their NOK (Mdn = 65.0, IQR = 57.5–90; *p* = 0.049).

Furthermore, *Family* was nominated in both patient’s self-rating as well as NOK’s estimation of the patient’s QoL (external rating) in n = 13 couples. For those couples, NOK significantly underestimated patients’ satisfaction (Mdn = 70.0, IQR = 60–80) compared to patients’ self-ratings (Mdn = 90.0, IQR = 60–80; *p* = 0.047). In total, 7 of the 13 NOK underestimated the respective patient’s satisfaction by an average of 25.7 points, while only one overestimated the patient’s satisfaction.

Regarding self-ratings of satisfaction for other frequently nominated areas, patients reported a very high satisfaction for *Marriage/Partnership* (n = 11, Mdn = 100, IQR = 90–100) and a moderate to high satisfaction for *Friends/Social Activities* (n = 10, Mdn = 75.0, IQR = 60–87.5). For *Physical Health*, the median of satisfaction indicated by patients as well as by NOK was on a moderate level, but with a remarkably higher range of ratings in patients (*n* = 11, Mdn = 50, IQR = 5–55) than in NOK (*n* = 8, Mdn = 60, IQR = 57.5–72.5).

### Depression and anxiety

**Fig. 4:**
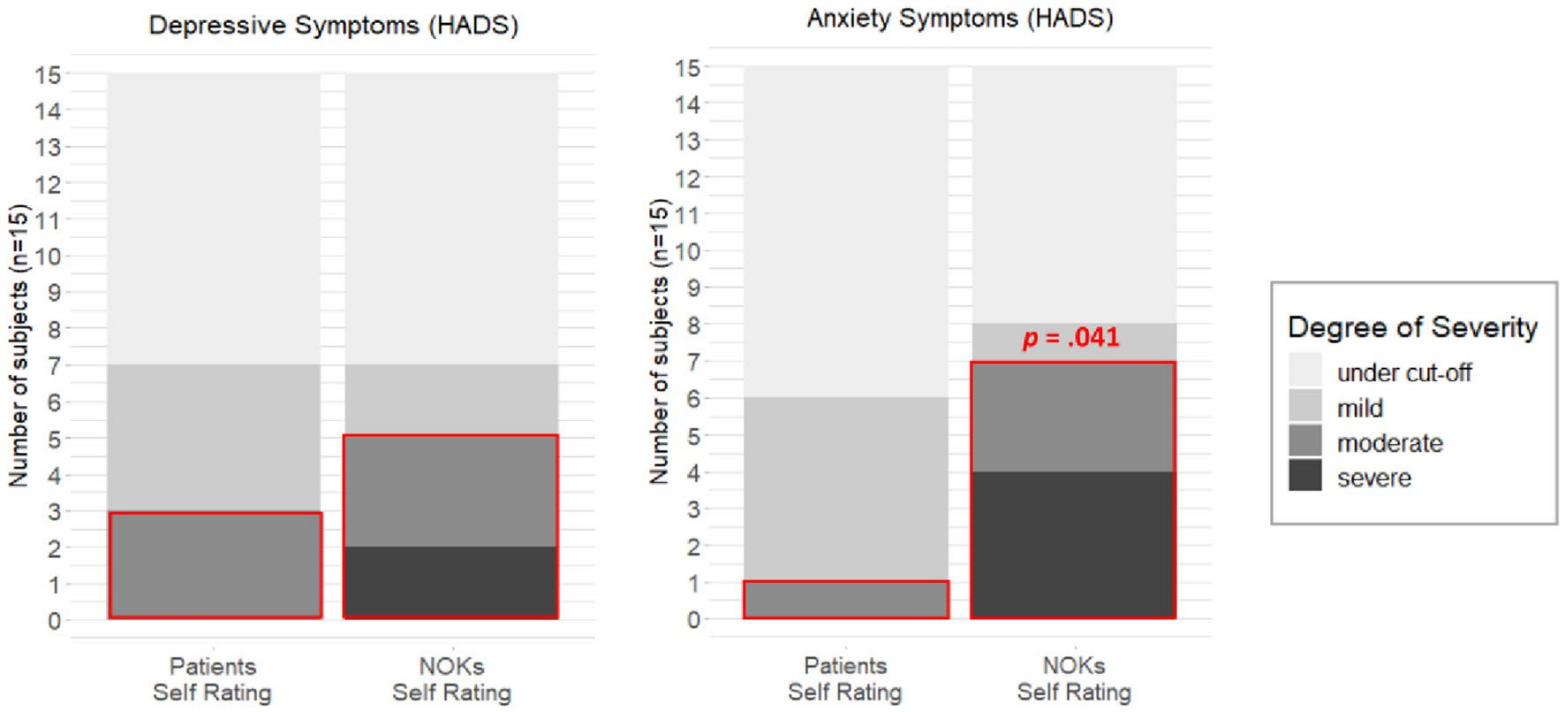
Severity levels of depressive and anxiety symptoms reported in the HADS. Red borders mark symptoms classified as clinically relevant (moderate to severe). Statistically significant results (*p* < 0.05) are reported for the comparison between patients and NOK regarding proportions of those with clinically relevant symptoms (McNemar´s Test)

Three iLIS patients (20%) indicated moderate and thus clinically relevant symptoms of depression, while five NOK (33.3%) reported clinically relevant symptoms on a moderate to severe level. Severity of depressive symptoms did not differ significantly between patients and NOK (Table 1, Fig. 4).

One iLIS patient (6.7%) indicated clinically relevant anxiety on a moderate level, whereas a total of seven NOK (46.7%) reported moderate or even severe and thus clinically relevant anxiety symptoms. Anxiety scores were significantly higher in NOK compared to patients (*p* = 0.015, see Table 1) and more NOK than patients indicated a clinically relevant manifestation of anxiety symptoms (*p* = 0.041; Table 1, Fig. 4).

The data revealed a strong relationship between mental health and QoL in patients: Higher depression and higher anxiety symptoms scores were both correlated with lower self-ratings of global QoL in the McGill-SIS (depression: *r* = − 0.73, *p* = 0.002; anxiety: *r* = − 0.81, *p* < 0.001) and of subjective QoL in the SeiQoL-DW (depression: *r* = − 0.56, *p* = 0.035; anxiety: *r* = − 0.72, *p* = 0.003). The corresponding analyses for NOK indicated significant correlations for depression and anxiety only for the McGill-SIS (depression: *r* = − 0.71, *p* = 0.003; anxiety: *r* = − 0.60, *p* = 0.02) and not for the SeiQoL-DW (depression: *r* = − 0.41, *p* = 0.14; anxiety: *r* = − 0.13, *p* = 0.64).

Examining the relationship between NOK’s own mental health and their evaluation of patients’ QoL, a higher depression symptoms score in NOK was moderately correlated with a lower external rating of global QoL-rating in the McGill-SIS (*r* = − 0.53, *p* = 0.043). Concerning NOK’s anxiety, there was no significant correlation, but a statistical trend (*p* < 0.10) for a negative association with NOK’s external rating of patient’s McGill-SIS-scores: NOK with higher anxiety scores tend to rate patients’ QoL lower (*r* = − 0.48, *p* = 0.068).

For the SeiQoL-DW, we observed no significant correlations between NOK’s depression or anxiety scores with their external ratings of patients’ subjective QoL (depression: *r* = 0.05, *p* = 0.86; anxiety: *r* = − 0.34, *p* = 0.24).

Of note, regarding patients’ estimation of their NOK’s QoL, neither patients’ depressive symptoms scores nor their anxiety symptoms scores were significantly correlated with their estimations of their NOK’s QoL in the McGill-SIS (depression: *r* = − 0.23; *p* = 0.40; anxiety: *r* = − 0.15, *p* = 0.60).

### Caregiver burden

The caregiver burden of NOK was on average on a moderate level (see Table 1), where three NOK (20%) indicated a low caregiver burden, six NOK (40%) reported a moderate caregiver burden and another six (40%) a high caregiver burden.

With respect to the importance of caregiver burden, its relationships with QoL and psychological wellbeing were analyzed. We obtained a moderate positive correlation between caregiver burden and anxiety symptoms score (*r* = 0.64, *p* = 0.01). NOK with clinically relevant (moderate to severe) anxiety symptoms reported a stronger caregiver burden (Mdn = 25.0, IQR = 24.0–25.5) than those with anxiety scores below the threshold for clinical relevance (Mdn = 11.50, IQR = 8.5–15.0; *p* < 0.001). NOK’s caregiver burden neither correlated with their depression symptoms (*r* = 0.35, *p* = 0.21) nor with their self-rated QoL (SeiQoL-DW: *r* = − 0.30, *p* = 0.29; McGill-SIS: *r* = − 0.28, *p* = 0.31).

Concerning again the interrelation between NOK’s own wellbeing and their estimation of patient’s QoL, a higher caregiver burden score was correlated with a lower rating of patients’ QoL in the SeiQoL-DW (*r* = − 0.63, *p* = 0.016). With respect to the McGill-SIS, there was no significant correlation between caregiver burden and NOK’s estimation of patients’ QoL (*r* = 0.39, *p* = 0.15).

### Association with sociodemographic and clinical characteristics

Sociodemographic, disease- and everyday life-referred characteristics were analyzed regarding their potential influence on QoL, psychological wellbeing and caregiver burden. Age was not significantly correlated with QoL, psychological health or burden in NOK or patients. For gender, the only significant difference were higher anxiety scores in female compared to male NOK (*p* = 0.03). None of those psychological measures differed with regard to ALS-onset or was associated with the time since ALS-diagnosis in patients or NOK.

Moreover, NOK were asked how many hours per day they usually spend with the patient. Of note, a higher number of hours showed moderate correlations with a higher self-reported QoL in the McGill-SIS (*r* = 0.56, *p* = 0.029), with lower anxiety (*r* = − 0.52, *p* = 0.044) and with lower depressive symptoms scores (*r* = − 0.54, *p* = 0.038) in NOK. For iLIS patients, this number of hours spend together showed no significant correlation with any of the psychological self-report measures.

### Attitude toward invasive ventilation

Eleven patients used invasive ventilation (IV) at the time of the study visit, almost all of them for 24 h a day. Five patients began to receive IV following an emergency situation (hypercapnia), but were still able to give their consent prior to the procedure. One patient received IV in an emergency procedure without his consent or knowledge of his regarding attitude or wish. In two cases, patients had to decide about IV at short notice (less than 2 weeks) due to their critical respiratory state. Three patients planned initiation of IV long-term beforehand. Of the 11 patients with IV, 9 stated that they would choose this treatment again while the other 2 answered that they would not.

## Discussion

While QoL, mental health and caregiver burden have been investigated intensively in early to middle stage ALS, there is little research on patients and even less on their NOK in advanced disease stages. While some of this previous research suggest quite high QoL for patients compared to a lower QoL for the NOK, we still know little about differences and interaction between patients’ and NOK’s QoL, mental health and burden and by which factors they might be influenced—particularly for iLIS, the stage in which ETCS technology is needed to capture patients’ perspective in a direct and independent manner. To close this research gap, we screened 52 patients with ALS in suspected iLIS and were able to investigate those clinically highly relevant aspects in 15 iLIS patients-NOK-couples, using ETCS for patients’ assessment.

Patients’ QoL was on a moderate level and similar to QoL-ratings by NOK—including NOK’s self-ratings as well as their estimations of patient’s QoL. Thus, no significant misjudgment by NOK was evident, whereas patients overestimated their NOK’s QoL. Prominent findings were made for anxiety: most importantly, more severe anxiety symptoms were reported by NOK than by iLIS-patients. Higher anxiety in NOK was moreover associated with higher caregiver burden and lower QoL. Regarding the key issue of NOK’s estimation of iLIS patients’ QoL, this estimation was significantly associated with NOK’s own depressive symptoms and caregiver burden—pointing to an influence of NOK’s own wellbeing on their perception of patient’ QoL or vice versa. Importantly, while NOK’s anxiety, depression and QoL depended strongly on the time they spent with the patient, this was not the case for the iLIS patients themselves.

An average moderate subjective QoL is in line with findings for less advanced ALS patients [13, 15, 17, 49] and with a recent study for ALS-patients in iLIS [25]. The great majority of present iLIS patients reported a good QoL, particularly in the SeiQoL. This confirms the “wellbeing paradox” in ALS [50] as the effect of an effective adaption to the *objectively* severe limitations of physical abilities and thus everyday life due to the disease. Apparently, the ability to adapt even allows a satisfying life in iLIS and thus in a state where those limitations peak.

Regarding this conclusion, lower QoL-ratings in the McGill-SIS compared to the SeiQoL-DW need to be considered though. On one hand, this discrepancy might challenge the validity of the questionnaires as measuring instrument for subjective (global) QoL. On the other hand, the differences suggest that SeiQoL-DW and McGill-SIS assess deviating concepts of QoL. This is an important issue, since both questionnaires are popular and often used in ALS research, but rarely together. One of those few studies [51] found comparable high QoL-ratings in McGill-SIS and SeiQoL-DW in ALS-patients, but a weak correlation between those ratings. The authors concluded that the SeiQoL measures various QoL-related concepts such as “happiness”, while the McGill-SIS is a valid measure of global QoL – confirmed by its strong correlation with more complex and also ALS-specific measures of global QoL. In contrast, we observed a strong correlation between iLIS patients’ self-ratings in McGill-SIS and SeiQoL-DW, suggesting a rather (linear) shift of QoL-ratings instead of indicating the measurement of different constructs.

Present results for the areas of life nominated in the SeiQoL-DW might provide an important explanation for this displacement: social areas (family, marriage/partnership, friends) were chosen very frequently. Almost all iLIS patients selected at least two of the three, most often family and marriage with consistently high satisfaction ratings. This is in line with the finding that non-depressive patients—as the great majority of the iLIS patients—mainly nominate areas that are not strongly compromised by their disease [2, 17]. It can be concluded that patients’ high satisfaction with their social relations influenced their QoL-ratings in the SeiQoL-DW much stronger than in the McGill-SIS. Based on our findings, we therefore postulate that the McGill-SIS provides a rather comprehensive or *global* view on QoL, which is more strongly based on objective criteria and consequently evokes lower ratings by iLIS-patients as it is the case for the SeiQoL-DW. This is further supported by a longitudinal stability of ALS-patient’s QoL in the SeiQoL-DW [10, 13, 31] and it’s lacking correlation with the degree of motor impairment [13, 18, 38], while McGill-SIS-scores were shown to decrease and to be associated with the ALSFRS-R [52]. Although we did not find a similarly strong relationship between ratings in SeiQoL-DW and McGill for the NOK, there is a clear trend in the same direction, which needs to be interpreted with regard to the small sample size.

Regardless of the respective extent of patient’s QoL, the SeiQoL-DW-results for areas of life provide valuable information about factors influencing patients’ QoL and wellbeing [51]. Nominated areas of life prove that social relations and probably the social support provided by them are the most important resource for a satisfying subjective QoL in advanced ALS. This was previously shown for patients in earlier stages [13, 15, 31, 49] and in iLIS [24]. Furthermore, family was named very often in several of those studies for earlier ALS [13, 28, 31, 49, 53] and rated highly satisfactory [15, 17]. However, the particular importance of the marital relationship has been rarely reported before [15].

The finding of a mostly moderate to high subjective QoL of ALS patients—that remains stable albeit the progressing motor function impairment—is explained by a psychosocial adaption process. Discussed as an important mechanism of this adaption is the increasing relevance of social relations and “internal” aspects (like mental health), while the importance of “external” and other usually compromised aspects like occupation, finances as well as physical health decrease [13, 15, 21, 54]. This process is assumed to reflect a “frame shift” [55]: an internal readjustment of values, expectations and priorities, forming the basis of patients’ perception of their QoL and evaluation of a life worth living. It results in higher congruence of those internal standards with the changing reality and remaining possibilities and therefore in maintenance of (good) subjective wellbeing or QoL [13, 17, 24]. Our SeiQoL-results support that such a process can proceed until iLIS, although capabilities and possibilities for an active social life are severely restricted at this stage. Compared to earlier ALS, iLIS is characterized by essential limitations of autonomy and participation. However, this stage often goes with a better professional support system (e.g., 24-h nursing service) and is characterized by stability instead of the ongoing worsening and losses of functions at earlier ALS stages. This can be assumed to facilitate adaption and thus promote wellbeing and QoL, together with a longer disease duration per se as a key factor for psychosocial adaption in ALS [26]. An important implication of those results is the crucial role of ETCS-supply for QoL [56–59] since ETCS remains the only mean for complex communication and active social participation in iLIS. The essential significance of this technology is underlined by the extensive use of iLIS-patient’s personal ETCS for more than 10 h daily on average.

The negative correlation between QoL and severity of depressive symptoms for iLIS patients is in concordance with findings for earlier ALS [1, 33, 60, 61]. With 20% experiencing clinically relevant symptoms and a mean score below the threshold for mild symptoms, the extent of depression was even lower than in a study of earlier ALS patients using the HADS [46] and only slightly above the mean HADS-score of 5.9 reported in a review of depression in ALS [62]. A recent study of polish ALS patients in iLIS reported a marginally higher prevalence of 26% of clinically relevant depression symptoms in the ADI [25]. The ADI questionnaire is similar to the HADS by excluding somatic symptoms, thereby delivering quite comparable results for ALS patients [46]. Of note, however, nearly half of iLIS-ALS patients but only 4% of NOK in our study were treated by antidepressants. While this might reduce depressive and also anxiety symptoms in patients and thus partly explain differences to their spouses, it underlines the unmet need for specific support for the NOK of ALS patients.

Anxiety in ALS has been studied less often than depression [23] and to the best of our knowledge never before for iLIS. Clinically relevant (moderate) anxiety symptoms were less prevalent than depression symptoms (6.7%) and even below the recently reported low rates between 12 and 15% in earlier ALS [13, 22]. This might seem surprising, considering what could cause anxiety in iLIS patients: a complete loss of mobility and their abilities to speak, to breathe, resulting in a dramatic loss of autonomy and control [54, 63]. It was however shown before that increased anxiety arises in reaction to the ALS-diagnosis, but diminishes in the course of the disease [64, 65]. In accordance, ALS patients report worries about upcoming symptoms and deterioration and particularly respiratory failure as strongest causes of fear and anxiety [54, 63, 66]. These concerns might not to exist anymore at the severe but stable stage of iLIS.

From the perspective of a healthy person, an iLIS-patient’s situation appears hopeless and therefore inevitable to result in despair, strong fear and depression [26]. The present results, however, demonstrate once more that a relevant part of ALS patients maintains a good mental health, even or maybe particularly in advanced ALS. Nevertheless, some of the participating iLIS patients experienced serious depressive and anxiety symptoms and these symptoms were strongly associated with a lower subjective QoL. Results thereby confirm that depression and anxiety are no very common phenomena in ALS, but need to be diagnosed and if apparent, treated appropriately [61]. This is often neglected in clinical practice [67], probably because depression and mental health issues are misjudged as a natural and thus usual or even inevitable consequence of this severe and fatal illness [61].

Research revealed that even ALS patients’ closest relatives underestimate their loved one’s psychological wellbeing and QoL [1, 24, 26], including our own previous and first eye tracking-based study of ALS-patients in iLIS (using the SeiQoL-DW as only measure of QoL). Evidence is not consistent though: in concordance with our present results, no reliable underestimation was found in another sample of German ALS patients [38] as well as for polish ALS patients in iLIS [25]. In contrast to our preliminary ETCS-based study, current results show frequent underestimations as well as overestimations of QoL. This deviation can be mainly attributed to patients’ remarkably lower QoL-self-ratings in our current compared to the previous sample, in which they were homogenously very high (mean of SeiQoL-DW-Score around 81) – while NOK’s estimations of their own and patients’ QoL were on a very similar level in both studies.

As an important explanation, we consider recent findings to be less biased by a selection of iLIS patients with an above average subjective wellbeing and therefore to be more representative of the  iLIS population. This is supported by the larger sample size and greater heterogeneity of patients’ characteristics (e.g., ALS- and IV-duration, place of living). In further comparison to our first iLIS study, the present subset of iLIS-patients whose QoL in the SeiQoL-DW was underestimated by their NOK reported a comparable, very high QoL (mean around 77). This points to the existence of a subgroup of patients with a very high and crucially underestimated global QoL—which we selectively looked at in our first study and in the present one alongside to patients with moderate and even low QoL.

Importantly though, we still see an—often strong—underestimation of iLIS-patient’s QoL by their spouses in at least half of the couples, not surprisingly to a greater extent for the SeiQoL-DW in which patients rated their QoL higher than in the McGill-SIS.

Comparing the important areas of life reveals that those underestimations largely reflect a particular underestimation of the value of social relations: spouses remarkably underrated patients’ satisfaction with their family as well as the importance of their marital relationship with the patients for their subjective QoL. This specifies the underestimation of the importance of social activities which we observed in our previous study. Although causal conclusions are not possible, the present results suggest that this misjudgment is partly attributed to NOK’s own lower satisfaction with those social aspects of life.

Misjudgments of patients’ QoL are particularly important because of NOK’s well known impact on patients’ medical decisions including those over life maintaining measures [16, 38]. The great majority of patients do not want to take these decisions on their own, but together with their health professionals and family [39]. Therefore, the perception of the patient’s wellbeing by other family members—which might be influenced by the impairment of NOK’s own QoL and their caregiver burden—is of essential importance in the regard of fulfilling patient’s actual will and their safety.

Regarding our central hypothesis of an interaction between NOK’s own—compromised—wellbeing and their estimation of the patient’s QoL [24], the present study provides supportive evidence particularly for depressive symptoms and caregiver burden: NOK with more severe symptoms and burden reported lower estimations of patients’ QoL. On one hand, this suggests stronger (psychological) strain as risk factors for underestimating iLIS patients’ QoL. In fact, a selective, negatively biased perception and interpretation of emotions and situations are core characteristics of depression. A depressive mood most likely influences how the NOK evaluates their spouse’s QoL. On the other hand, it can also be presumed that the NOK’s assumption of a poor QoL of their partner enhances depressive symptoms and burden. NOK’s dedication and effort to provide their beloved ones with the best possible wellbeing puts a great strain on their psychological, physical and time resources. To assume that the patient experiences a poor QoL anyway, could obviously cause or intensify depressive symptomatology and burden. Supporting that, NOK particularly underestimated patients’ appreciation and satisfaction with family and with their marital relationship in the SeiQoL and thus their *own* value for patient’s QoL.

Caregiver burden was mostly moderate to high, concordant with findings for NOK of less advanced ALS patients [29, 68]. Unlikely those earlier ALS patients though, almost all of the iLIS patients were taken care of by professional caregivers around the clock. This matches what we learned in our study interviews with the NOK as well as in clinical practice: while the professional care is indispensable, NOK are still highly demanded by patient’s nursing, care and organizational matters. In case of patients who are taken care for at home, a great burden for their spouse emerges from lacking personal space and time and always being “on duty.” For NOK of patients in a nursing home, emotional strain is caused e.g., by experiencing guilt and sadness for not being able to share a home and not being around all the time. NOK are generally torn by conflicting needs: spending as much of the remaining time as possible with the patient and ensuring their best possible support on one side, and having a life outside their caregiver role on the other side. SeiQoL-DW-results indicate that the resulting psychological burden was often reinforced by financial worries relating to their spouses’ disease. The result of strong caregiver burden in NOK of iLIS patients is in line with evidence for an increasing burden with increasing ALS severity [29].

In view of this physical and psychological exhaustion, it is not surprising but relevant that NOK who were more severely burdened by their caregiving duties also indicated stronger anxiety. Together with the high prevalence of clinically relevant anxiety symptoms in nearly half of the NOK, this underlines the importance of not only practical but also psychological support for iLIS patient’s NOK, within the framework of specialized multidisciplinary care in ALS. Such support for NOK is explicitly recommended in the treatment guidelines for ALS [69], but not yet established in clinical practice. So far, anxiety has been hardly studied in ALS and even less for the NOK than for the patients. Present results point to the need to pay more attention to this aspect of mental health, e.g., regarding sources and contents of anxiety also to derive specific measures and (psychological) interventions. Similar to patients, clinically relevant depression symptoms were less frequent but still affected one third of the NOK and were—as well as anxiety—associated with a lower QoL.

As one specific aspect of burden and restriction of their wellbeing and QoL, NOK rarely named their marriage/partnership as an important area of life, while it was such a crucial source of QoL for the patients. Olsson et al. [15] made a similar finding for earlier ALS patients and their spouses. We presume it to reflect a subjective, hurtful experience of a loss of their partnership as it existed before the onset of the disease. This is probably associated with a change from partner to caregiver or the overlapping of those roles [70, 71] and patients’ severely restricted possibility to play an active role in the relationship. Regarding one facet of the changing relationship, Sandstedt et al. reported that iLIS-patient’s NOK indicate the least satisfaction with the QoL-domain of sexual life [72]. However, this subject has been widely neglected in research to date. In sum, the present results for advanced ALS appear to support that NOK compared to patients adapt less successfully to the disease and underlying experience of loss (e.g., of partnership, time for friendships and leisure activities) [73].

The proportion of 80% of the LIS-patients who would choose the crucial life prolonging measure of IV again is almost exactly as high as for patients with shorter ALS-duration and still better motor functioning [26]. It is also concordant with the finding that 10–20% of ALS patients report a wish to die [74, 75]. An important implication is that even though a small proportion of patients choose IV [76], those who do are mostly satisfied with this decision – as we can show here, also at the most advanced stage of the disease. In accordance, patients report depressive symptomatology on a low level. These are important findings considering that physicians overestimate the negative effect of IV on patients’ QoL [77]. Therefore, the current findings underline the importance of physicians to communicate directly with their patients in order to learn about their wellbeing and (life critical) wishes at each stage of the disease. This essential need for communication despite of the strongly limited communication abilities must be fulfilled e.g., by using ETCS.

The main limitation of the present study is its small and still selective sample, restricting the generalizability of results for the population of iLIS patients and their NOK. With regard to this positive selection, all included patients were willing to participate in a time-consuming and demanding psychological study. All patients not only lived in a partnership but most of them had a larger and supporting social network, as underpinned by the SeiQoL-DW-results. Moreover, they were all supplied with a personal ETCS, since this is covered by health insurance in Germany. The existence of a positive bias is further supported by the quite high number of screening fails and by frequent reasons for dropping out of the study, mainly a compromised state of physical and also explicitly mental health as well as oculomotor and cognitive deficits that do not allow or limit the use of an ETCS. Comparing characteristics of included patients with those who rejected study participation, dropped out or were excluded from analysis in fact discloses differences regarding, e.g., living in a partnership/marriage, use of personal ETCS and of, IV as well as duration of ALS; differences that need to be considered as influencing factors on QoL and mental health and therefore as a limitation of the study results.

Nevertheless, we present data for a more heterogeneous and thus more likely representative sample than comparable studies [24, 25], including amongst other things a quite high proportion of approx. 30% of iLIS patients who live in a nursing home. Importantly, we identified potential reasons for differing QoL and well-being of iLIS patients and their NOK as well as misjudgments of each other´s QoL. Noteworthy is the overestimation of NOK’s QoL by the patients on the one side and the high levels of depression and particularly anxiety in NOK but not in iLIS patients on the other side. Nevertheless, longitudinal studies with larger sample sizes are needed to validate our findings, although difficult to carry out due to the rarity and severity of the condition. In this regard, relatively small samples, also due to high drop-out-rates, need to be seen as a general limitation of research in this field and this specific patient population.

## Conclusions

Taken together, the present findings for advanced ALS show that direct assessment by ETCS is possible in iLIS. The high associations between QoL and wellbeing-measures in patients confirm validity of developed eye tracking versions of the psychological questionnaires. Although IV was mainly initiated as emergency measure or short term decision, many ALS-LIS patients presented with mostly good QoL and would choose this measure a second time, if they were faced with this decision again.

NOK are severely burdened, possibly influenced by or vice versa influencing their perception of patients QoL. Compared to the patients, NOK appear to psychologically adapt less successfully to the disease and associated experiences of loss (e.g., of partnership, time for friendships and leisure activities) in contrast to the frame shift on the side of the patients [73]. This might particularly lead to strong anxiety and moreover increase the risk of underestimating iLIS patients’ QoL. Intriguingly, while NOK’s anxiety, depression and QoL depended strongly on and correlated negatively with time spent with patients, this was not the case for the patients themselves. These data point to dramatic need of support in the strengthening of the relationship between iLIS patients and their respective NOKs.

## Supplementary Information

Below is the link to the electronic supplementary material.

Supplementary file1 (PDF 310 kb)
