# Supplementary material for: Quality of life and mental health in the locked-in-state—differences between patients with amyotrophic lateral sclerosis and their next of kin
Source: J Neurol. 2022 Jul 6;269(11):5910–25. doi: 10.1007/s00415-022-11238-0 (PMC9553779; doi:10.1007/s00415-022-11238-0)
Supplement: Supplementary file 1 — Supplementary file1 (PDF 310 kb) [file 415_2022_11238_MOESM1_ESM.pdf]

Title Quality of life and mental health in the locked-in-state–differences between patients with amyotrophic lateral sclerosis and their next of kin

Elisa Aust[1], Katharina Linse[1,2], Sven-Thomas Graupner[3], Markus Joos[4], Daniel Liebscher[4], Julian Grosskreutz[5], Johannes Prudlo[6,7], Thomas Meyer[8], René Günther[1,2], Sebastian Pannasch[3], Andreas Hermann[7,9,10]*

[1] Department of Neurology, Technische Universität Dresden, Dresden, Germany
[2] Deutsches Zentrum für Neurodegenerative Erkrankungen (DZNE) Dresden, Dresden, Germany
[3] Engineering Psychology and Applied Cognitive Research, Technische Universität Dresden, Dresden, Germany
[4] Interactive Minds Research, Interactive Minds Dresden GmbH, Dresden, Germany
[5] Precision Neurology, University of Lübeck, Lübeck, Germany
[6] Department of Neurology, University of Rostock, Rostock, Germany,
[7] Deutsches Zentrum für Neurodegenerative Erkrankungen (DZNE) Rostock/Greifswald, Rostock, Germany
[8] Center for ALS and other Motor Neuron Disorders, Charité – Universitätsmedizin Berlin, Corporate Member of Freie Universität Berlin, Humboldt-Universität zu Berlin, and Berlin Institute of Health, Berlin, Germany
[9] Center for Transdisciplinary Neurosciences Rostock (CTNR), University Medical Center Rostock, University of Rostock, Rostock, Germany
[10] Translational Neurodegeneration Section “Albrecht Kossel”, Dept. Neurology, University of Rostock, Rostock, Germany

***Corresponding Author:**
Prof. Dr. Dr. Andreas Hermann
Schilling Professor for Translational Neurodegeneration
Translational Neurodegeneration Section "Albrecht Kossel"
Dept. Neurology
University Medical Center Rostock
Gehlsheimer Straße 20, 18147 Rostock, Germany
Phone: +49 (0)381 494-9541
Fax: +49 (0)381 494-9542
eMail: Andreas.Hermann@med.uni-rostock.de

SUPPLEMENT

Table S.1 Demographic and clinical characteristics of excluded patients (screening fails, rejecters, drop-outs and patients who were excluded because no NOK participated in the study)

Characteristic	Screening Fails	Rejecters	Drop-outs	Participation without NOK
n	9	7	6	8
Gender, Female:Male,%	60:40	57.1:42.9	50:50	62.5:37.5
Age, yr[a],b	-	-	60.5 [56.75–65.0]	57.0 [51.75–65.25
Married/in Partnership,%	88.8	71.4	66.7	50
Education, yr[a,b]	-	-	16.0 [13.0–18.0]	15.0 [13.0– 17.25]
Working activity,% Employed:Retired or unemployed	-	-	-	25:75
Place of living, Home:Nursing home,%	88.8:12.2	71.4:29.6	67.7:33.3	62.5:37.5
Antidepressant medication,%[b]	-	-	60	62.5
ALS onset, bulbar:spinal,%[b]	-	-	33.3:66.7	0:100
ALS duration, yr[a,b]	-	-	7.25 [6.6–8.2]	9.3 [3.4–11.9]
ALSFRS-R[a,b]	-	-	1.0 [1.0–1.0]	2.0 [1.0 – 3.0]
IV,%; duration, yr[a,b]	44.4; -; -	71.4; -; -	100; 54.6 [28.0–60.0]	100; 64.5 [16.75–85.25]
Use of personal ETCS,%, duration, yr; use h/dy[a,b]	44.4; -; -	71.4; -; -	; -; -	100; 4.3 [0.8–7.3]; 13.5 [9.75–16.25]

NOK Next of kin, *ALS* Amyotrophic Lateral Sclerosis, *ALSFRS-R* ALS Functional Rating Scale Revised, *IV* invasive ventilation

[a] Data presented as median [interquartile range: Q1–Q3]
[b] Data only available for subgroup of patients who completed the study procedure, but were excluded from analysis because no NOK participated
[c] Data available for n=7 patients
